# A New Shear Strength Criterion for Rock Masses with Non-Persistent Discontinuities Considering the Nonlinear Progressive Failure Process

**DOI:** 10.3390/ma13214694

**Published:** 2020-10-22

**Authors:** Bowen Zheng, Shengwen Qi, Songfeng Guo, Xiaolin Huang, Ning Liang, Yu Zou, Guangming Luo

**Affiliations:** 1 Key Laboratory of Shale Gas and Geoengineering, Institute of Geology and Geophysics, Chinese Academy of Sciences, Beijing 100029, China; zhengbowen@mail.iggcas.ac.cn (B.Z.); guosongfeng@mail.iggcas.ac.cn (S.G.); huangxiaolin@mail.iggcas.ac.cn (X.H.); liangning@mail.iggcas.ac.cn (N.L.); zouyu@mail.iggcas.ac.cn (Y.Z.); luoguangming@mail.iggcas.ac.cn (G.L.); 2 Innovation Academy for Earth Science, Chinese Academy of Sciences, Beijing 100029, China; 3 College of Earth and Planetary Sciences, University of Chinese Academy of Sciences, Beijing 100049, China

**Keywords:** non-persistent discontinuities, connectivity rate, direct shear test, progressive failure, strength

## Abstract

The shear strength characteristics of rock masses containing non-persistent discontinuities are strongly affected by discontinuities and rock bridges. The linear Jennings criterion cannot reflect the nonlinear mechanical behavior during progressive failure of rock masses with non-persistent discontinuities. In this study, a new nonlinear shear strength criterion was developed. First of all, a series of shear test data about artificial rock mass samples were collected on the basis of the published literatures, and five types of samples were differentiated according to the positions of discontinuities. After that, a new nonlinear shear strength criterion was proposed by introducing two correction coefficients *A* and *B* into the basic form of the Jennings criterion, which could correct the weight of the cohesion and the internal friction coefficient of rock bridges respectively. Then, the new criterion was determined by fitting the basic form of the Jennings criterion with the laboratory data. It was found that the parameters *A* and *B* had a nonlinear exponential and negative exponential relation with the connectivity rate respectively. It indicated that both the cohesion and the internal friction coefficient estimated by the new criterion were superior to those estimated by the Jennings criterion. Compared with the linear Jennings criterion, the new nonlinear shear strength criterion had a better applicability.

## 1. Introduction

The discontinuities always play an important role in the deformation, failure and mechanical behavior of a rock mass. Therefore, the mechanical characteristics—especially the shear properties of discontinuities—were researched by scholars from rock mechanics and engineering geology fields for the past decades [1,2,3]. For example, Barton (1973) [4] established a shear strength criterion for rock joints. Jing et al. (1993) [5] proposed a constitutive model for rock joints under cyclic shear loads. Homand et al. (2001) [6] explored the degradation of rock joints on the shear condition. On these bases, Qi et al. (2010) [7] and Saroglou et al. (2019) [8] evaluated the influences of discontinuities on the stability of slopes and tunnels under the gravity respectively. Except for the static gravity condition, previous studies were also focused on the effects of discontinuities on the dynamic stability of slopes under the seismic loads [9,10].

According to the persistent condition of discontinuities in a rock mass, the discontinuities can be divided into two types: persistent discontinuities and non-persistent discontinuities. A rock mass containing non-persistent discontinuities possesses both discontinuities and rock bridges [11]. Based on direct shear tests performed in laboratories, a number of researchers have conducted studies on such types of rock masses through the laboratory test, the numerical simulation as well as the theoretical analysis for the past decades [12,13,14]. For instance, Bai et al. (1999) [15], Chen and Tang (2008) [16], Liu et al. (2008) [17], Hu et al. (2008, 2011, 2012) [18,19,20] and Zhou et al. (2015) [21] conducted the studies about the influences of positions and connectivity rates of discontinuities on the shear characteristics for rock masses containing coplanar and non-persistent discontinuities. Considering the same position and connectivity rate of discontinuities, Liu et al. (2007, 2014) [22,23], Xia et al. (2010) [24], Tang et al. (2011, 2012) [25,26] carried out the researches about the effect of joint roughness on the shear properties of rock masses with coplanar and non-persistent discontinuities. Besides, Gehle and Kutter (2003) [27] and Gerolymatou and Triantafyllidis (2016) [28] explored the influences of dip angles for intermittent joints on the shear behavior of the rock mass.

Among previous researches Jennings (1970) [12] stated that the shear strength criterion could be acquired by the weighted average values of the shear strength parameters of discontinuities and rock bridges according to the connectivity rate of those discontinuities, and developed a Jennings criterion which has been widely used to quantitatively depict the shear strength of rock masses with non-persistent discontinuities. The Jennings criterion [12] is shown in Equation (1). It can be seen that the shear strength of a rock mass can be estimated through the Jennings criterion with a hypothesis that the rock mass strength has a linear relation with the connectivity rate.

(1) $$\tau =c+\sigma_{n}\tan\varphi =kc_{d}+\left(1-k\right)c_{r}+\sigma_{n}\left[k\tan\varphi_{d}+\left(1-k\right)\tan\varphi_{r}\right],$$

where *τ* is the peak shear strength; *σ*_n_ is the normal stress; *k* is the connectivity rate; *c*, *c*_d_, and *c*_r_ denote the cohesion of a rock mass, discontinuities and rock bridges respectively; and *φ*, *φ*_d_, and *φ*_r_ denote the friction angle of a rock mass, discontinuities and rock bridges respectively.

Although the Jennings criterion has been widely put into practices after being developed, it was always criticized because the assumption that the strength decreased linearly with the increase of the connectivity rate and thus could not reflect the real case [24,26]. Some researchers carried out modifications on the basis of the Jennings criterion. For example, Xia et al. (2010) [24] considered the roughness of discontinuities and the cohesion weakening of rock bridges. Tang et al. (2012) [26] took into account the concurrent weakening of both the cohesion and the internal friction coefficient of rock bridges. However, the modified Jennings criterions still could not characterize the nonlinear progressive failure process of a rock mass well.

A number of researchers have studied the progressive failure process of a rock mass under the static and dynamic load by the laboratory test and numerical simulation [29,30,31,32,33,34]. Among them Guo et al. (2017) [33] proposed a complete strength model, including both the shear strength and the tensile strength, entitled the CWFS-TL model based on the results of laboratory tests (Equation (2)). In their model, they stated that the cohesion strength weakened, the friction strength strengthened, the tensile strength lost as the damage increased, and quantitated the relations between the strength parameters with the plastic strain by nonlinear forms.

(2) $$\tau_{f}=\sigma_{n}\tan\varphi \left(\gamma_{p}\right)+c\left(\gamma_{p}\right),\;\sigma_{t}=\sigma_{t}\left(\varepsilon_{t}^{p}\right),$$

where $\varphi \left(\gamma_{p}\right)$ and $c\left(\gamma_{p}\right)$ denote that the shear strength parameters are functions of the plastic shear strain; $\sigma_{t}\left(\varepsilon_{t}^{p}\right)$ denotes that the tensile strength is the function of the plastic tensile strain.

Hence, the nonlinear mechanical behavior during progressive failure of rock bridges, which is vital for accurately estimating the shear strength of rock masses containing non-persistent discontinuities, should be considered. In this study, we attempt to propose a criterion to characterize the nonlinear shear behavior of rock masses containing non-persistent discontinuities with a data analysis method based on a series of available test data collected in the published articles.

The structure of this paper is as follows: a new nonlinear shear strength criterion was presented in the second section, and then the proposed criterion was verified based on a series of direct shear test results of rock masses containing coplanar non-persistent discontinuities in the third section. Some discussions and concluding remarks were presented in the fourth and fifth sections.

## 2. The Establishment of the New Shear Strength Criterion

In this section, a series of test data for rock masses with various discontinuity positions and connectivity rates were firstly collected from the published literatures. Then the way to establish the new shear strength criterion was presented and the final form of the criterion was proposed by fitting the test data.

### 2.1. Data Collection

**Figure 1:**
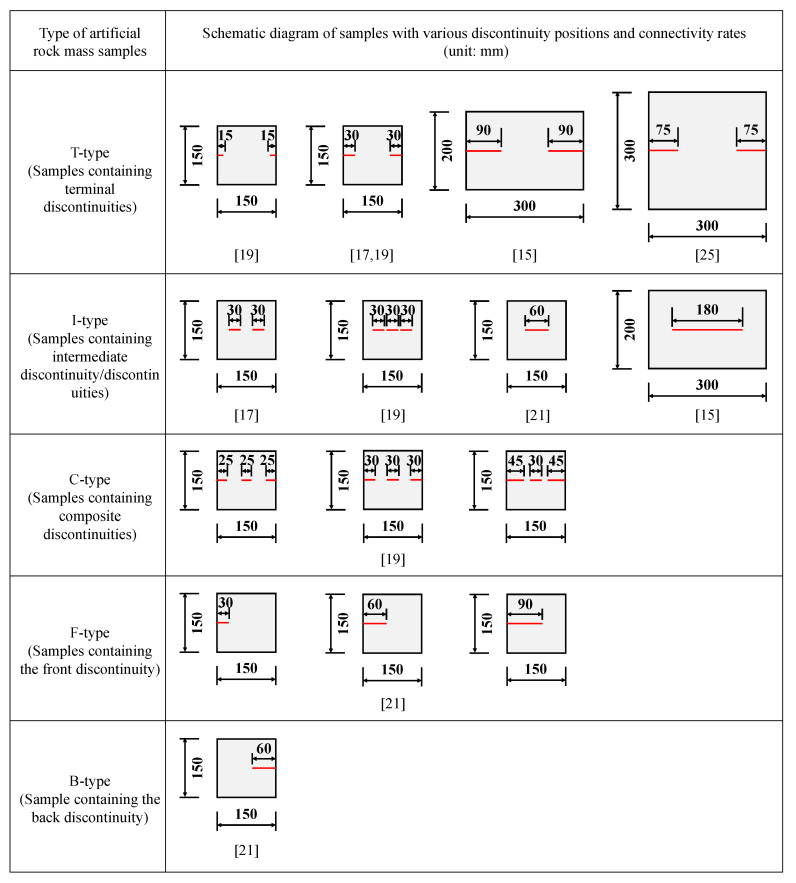
Schematic diagram of artificial rock mass samples with various discontinuity positions and connectivity rates.

Researchers have carried out numerous studies in laboratories mainly involving direct shear tests on rock or rock-like materials with non-persistent discontinuities. Due to the difficulties of sampling and processing natural rock mass samples with coplanar non-persistent discontinuities, artificial rock mass samples that were made of cement, sand, gypsum and other materials containing discontinuities with various positions and connectivity rates, have always been adopted. In spite of this, it was still not easy to manufacture the artificial rock mass containing discontinuities and control the shear loading process. In this paper, we collected sixteen groups of test data to establish the new shear strength criterion among the published papers, i.e., Bai et al. (1999), Liu et al. (2008), Hu et al. (2011), Tang et al. (2011), Zhou et al. (2015) [15,17,19,21,25]. Five types of samples with non-persistent discontinuities were differentiated, i.e., samples containing terminal discontinuities (T-type), samples containing intermediate discontinuity/discontinuities (I-type), samples containing composite discontinuities (C-type), samples containing the front discontinuity (F-type) and sample containing the back discontinuity (B-type) (Figure 1). The connectivity rate, material compositions as well as the strength parameters of both rock bridges and discontinuities are presented in Table 1.

The collected sixteen groups of direct shear tests were conducted on the artificial rock mass samples under constant normal load (CNL) conditions. The tangential load was exerted via the displacement or load control mode. Several normal loads were conducted to reach the strength parameters through Coulomb envelop lines. The shear rate and the strength parameters of samples with different types of discontinuities are shown in Table 2.

### 2.2. The New Shear Strength Criterion

As stated above, the cohesion and the internal friction angle of rocks have a nonlinear relation with the plastic strain [33]. It has been widely accepted that the increase of the plastic strain is resulted from the crack growth and coalescence [29,35,36,37,38]. Thus, the cohesion and the internal friction angle of rock bridges will be corrected by the connectivity rate in this study. Two correction coefficients *A* and *B* were introduced into the basic form of the Jennings criterion for the cohesion and the internal friction coefficient of rock bridges respectively. Then, the new shear strength criterion can be presented as Equation (3):

(3) $$\tau =kc_{d}+\left(1-k\right)Ac_{r}+\sigma_{n}\left[k\tan\varphi_{d}+\left(1-k\right)B\tan\varphi_{r}\right],$$

where *A* and *B* are dimensionless coefficients characterizing the effects of the connectivity rate on the cohesion and the internal friction coefficient of rock bridges respectively.

The correction coefficients *A* and *B* of the strength parameters can be acquired if the connectivity rate and the strength parameters of rock bridges, discontinuities as well as rock masses are given. The equations can be derived from Equation (3) and shown in Equations (4) and (5) respectively:

(4) $$A=\left(c-kc_{d}\right)/\left[\left(1-k\right)c_{r}\right],$$

(5) $$B=\left(\tan\varphi -k\tan\varphi_{d}\right)/\left[\left(1-k\right)\tan\varphi_{r}\right].$$

### 2.3. The Fitting Curve of Correction Coefficients Based on Test Results

According to Table 1 and Table 2 and Equations (4) and (5), the correction coefficients *A* and *B* of the strength parameters were calculated for rock mass samples with various discontinuity positions and connectivity rates respectively, as shown in Table 3.

**Figure 2:**
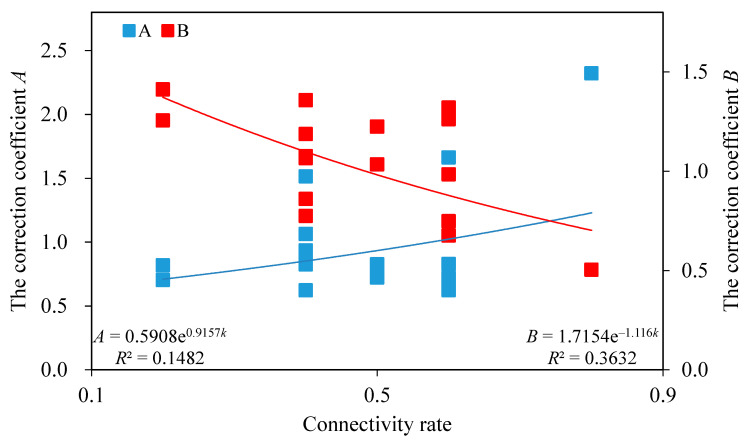
The correction coefficient *A* of the cohesion and the correction coefficient *B* of the internal friction coefficient for rock bridges with the connectivity rate.

From the data in Table 3, the correction coefficients *A* and *B* of the strength parameters were depicted for rock mass samples containing various discontinuity positions but the same connectivity rate, as shown in Figure 2.

As shown in Figure 2, once the connectivity rate increases, the correction coefficient *A* of the cohesion for rock bridges presents an increasing trend, while the correction coefficient *B* of the internal friction coefficient for rock bridges shows a decreasing trend.

Additionally, the exponential and negative exponential functions between the connectivity rate and the correction coefficients *A* and *B* were established respectively, which can be substituted into the new shear strength criterion in Equation (6):

(6) $$\tau =kc_{d}+0.5908e^{0.9157k}\left(1-k\right)c_{r}+\sigma_{n}\left[k\tan\varphi_{d}+1.7154e^{-1.116k}\left(1-k\right)\tan\varphi_{r}\right].$$

For a rock mass with undulating discontinuities, the parameter *φ*_d_ can be determined according to Equation (7) proposed by Barton (1973) [4]:

(7) $$\varphi_{d}=JRC\log_{10}\left(\frac{JCS}{\sigma_{n}}\right)+\varphi_{b},$$

where *JRC* is the roughness coefficient of discontinuities; *JCS* is the wall compressive strength of discontinuities; *φ*_b_ is the basic friction angle of discontinuities.

## 3. The Reliability of the New Shear Strength Criterion

Based on the Jennings criterion shown in Equation (1) and the new shear strength criterion shown in Equation (6), the estimated values of the cohesion and the internal friction coefficient for rock masses containing discontinuities with various positions and connectivity rates could be reached.

**Figure 3:**
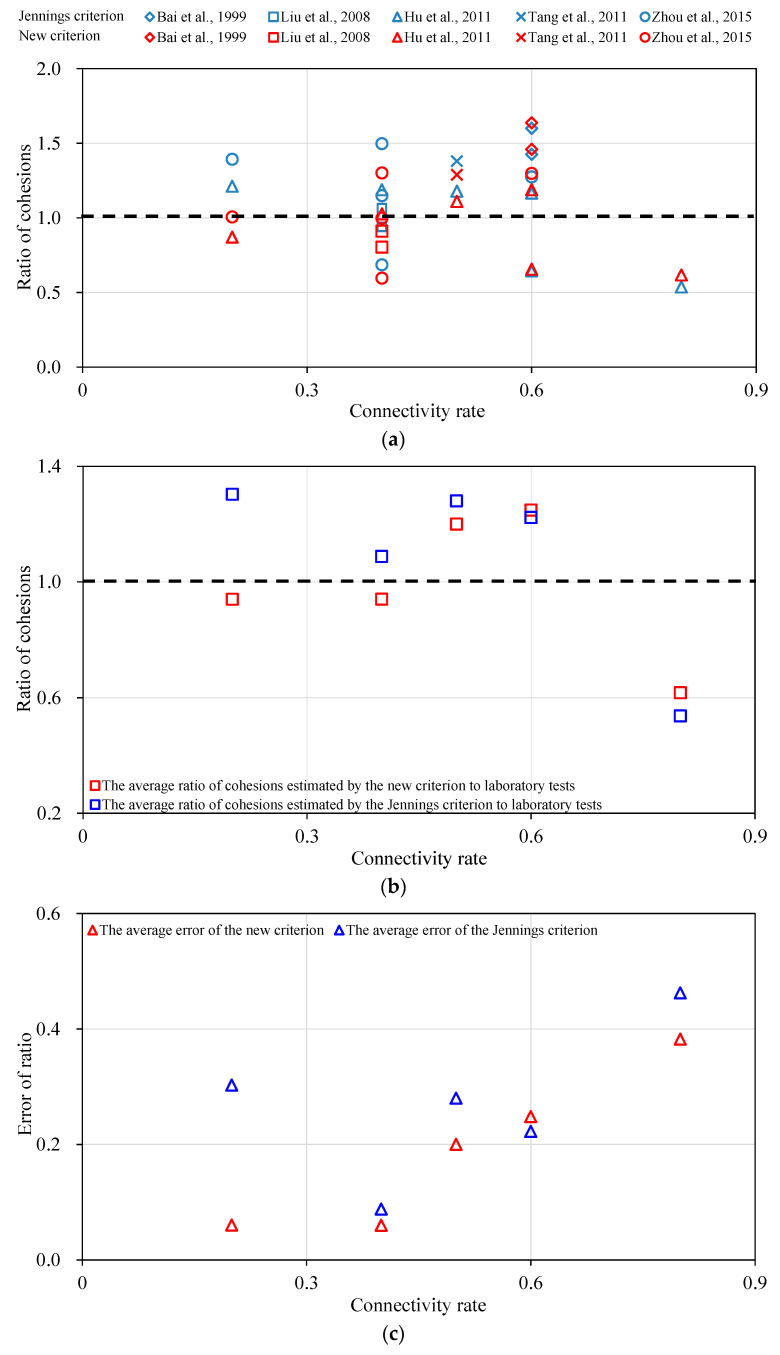
Comparison of the ratios of the estimated cohesions by the Jennings and new criterions to the test results of the cohesions with various connectivity rates. (**a**) The cohesion ratio with the connectivity rate; (**b**) The average cohesion ratio with the connectivity rate; (**c**) The error with the connectivity rate.

The connectivity rates as well as the strength parameters of the rock bridges and discontinuities for each group are given in Table 1, and thus the strength parameters of the rock mass samples with discontinuities can be estimated by both the Jennings criterion and the new criterion as shown in Equations (1) and (6) respectively. The comparison of the cohesions estimated by the Jennings and new criterions is presented in Table 4. The cohesions for different types of samples estimated by the criterions are shown as *c*_1_ and *c*_2_ in fourth and sixth columns respectively. And then the ratios of the cohesion estimated by the Jennings and new criterions to the real cohesion obtained by laboratory tests are shown as *R*_c1_ and *R*_c2_ in fifth and seventh columns respectively. It is obvious that the ratio of the estimated cohesion to the real cohesion can reflect the reliability of the criterions. Thus, we show these ratios of samples with non-persistent discontinuities in Figure 3a. The red color denotes the results estimated by the new criterion while the blue color denotes the results estimated by the Jennings criterion. The various shapes of labels denote the data from different references. The estimated result is better if the ratio is closer to 1. It can be seen that the estimated values by the new criterion are superior to those by the Jennings criterion for samples with connectivity rates of 0.2, 0.5 and 0.8. It is difficult to judge which is better for connectivity rates of 0.4 and 0.6. Therefore, we adopted a quantitative factor, i.e., the variance of the ratios to 1 (*V*^2^) (Equation (8)).

(8) $$V^{2}=\left[{\left(R_{1}-1\right)}^{2}+{\left(R_{2}-1\right)}^{2}+\cdots {\left(R_{n}-1\right)}^{2}\right]/n,$$

where *V*^2^ denotes the variance, *R*_n_ denotes the ratio and *n* denotes the number of data.

The new criterion can reflect the results from laboratory tests more correctly if the variance is smaller. The variances of cohesions estimated by the Jennings and new criterions are 0.12 and 0.08 respectively, which means that the latter one is superior to the former one obviously.

The average ratios of the cohesions for samples with the same connectivity rate are shown in Figure 3b, in which the red and blue squares denote the average ratios estimated by the new and Jennings criterions respectively. It can be seen that compared with the test results, the Jennings criterion overestimates the cohesions of test results in most cases, while the estimated results by the new criterion are a little smaller in three cases with connectivity rates of 0.2, 0.4 and 0.8 but a little larger in two cases with connectivity rates of 0.5 and 0.6. In general, the results reached by the new criterion are much closer to 1 than those by the Jennings criterion except for the samples with a connectivity rate of 0.6, under which condition the estimated result by the new criterion is quite close to that estimated by the Jennings criterion. It indicates that the new criterion is much better than the Jennings criterion.

The errors can be reached through calculating the differences between the ratios and 1, which are shown in Figure 3c. It indicates that the errors of results estimated by the new criterion are obviously much lower than those by the Jennings criterion except for the connectivity rate of 0.6. For the connectivity rate of 0.6, the average error of results estimated by the new criterion is a little larger, but quite close to that estimated by the Jennings criterion.

**Figure 4:**
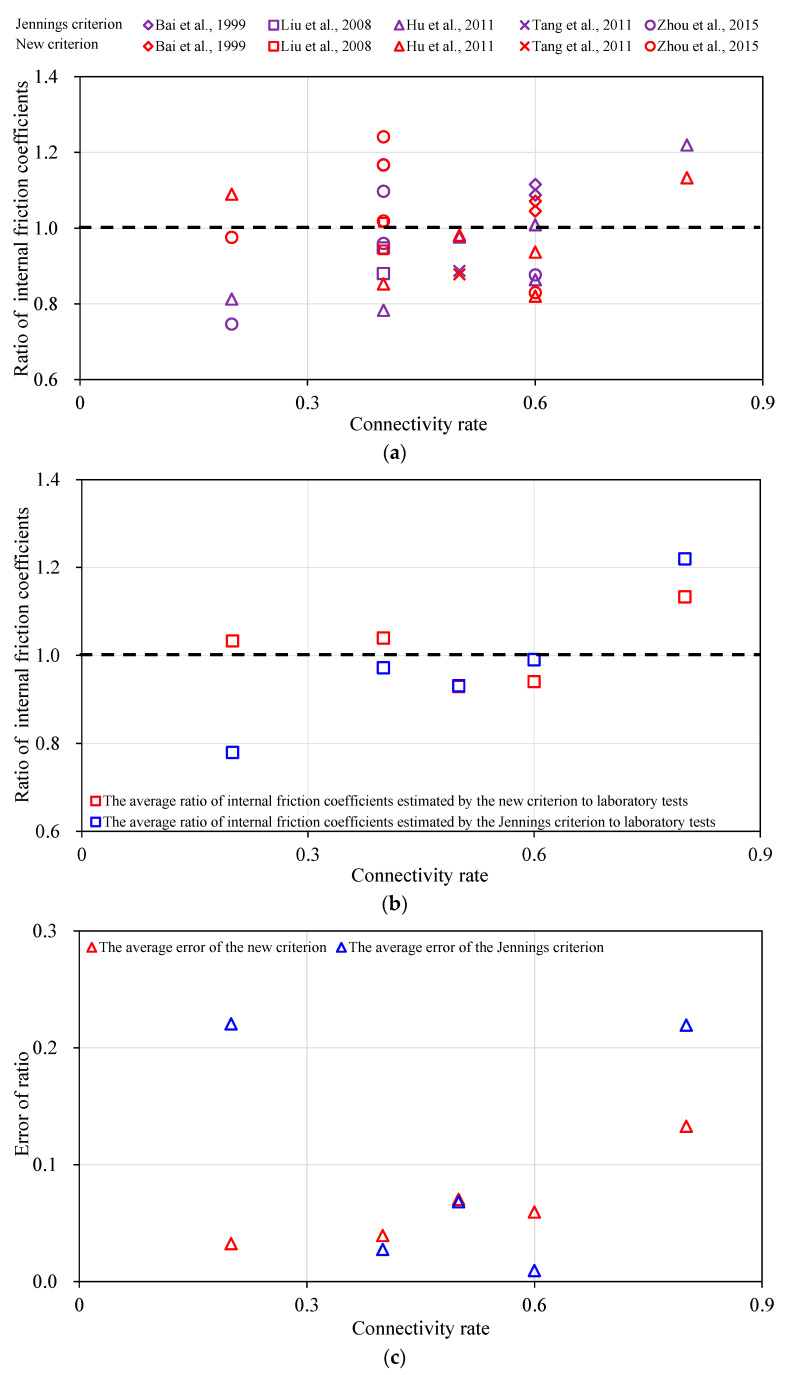
Comparison of the ratios of the estimated internal friction coefficients by the Jennings and new criterions to the test results of the internal friction coefficients with various connectivity rates. (**a**) The ratio of the internal friction coefficient with the connectivity rate; (**b**) The average ratio of the internal friction coefficient with the connectivity rate; (**c**) The error with the connectivity rate.

Similarly, the internal friction coefficients for different types of samples estimated by the Jennings and new criterions are shown as tan*φ*_1_ and tan*φ*_2_ in fourth and sixth columns respectively in Table 5. And then the ratios of internal friction coefficients estimated by the criterions to real internal friction coefficients obtained by laboratory tests are shown as *R*_f1_ and *R*_f2_ in fifth and seventh columns respectively. These ratios of samples with non-persistent discontinuities are shown in Figure 4a. The red color denotes the results estimated by the new criterion while the purple color denotes the results estimated by the Jennings criterion. The various shapes of labels denote the data from different references. It indicates that the estimated values by the new criterion are superior to those by the Jennings criterion for samples with connectivity rates of 0.2 and 0.8. It is difficult to judge which is better for connectivity rates of 0.4, 0.5 and 0.6. The variance is also adopted to judge qualitatively. The variances of internal friction coefficients estimated by the Jennings and new criterions are 0.02 and 0.01 respectively, which means the latter one is superior to the former one obviously.

The average ratios and errors of internal friction coefficients for samples with the same connectivity rate are depicted in Figure 4b,c, in which the red and blue squares denote the average ratios estimated by the new and Jennings criterions respectively. It is found that compared with the test results, the estimated internal friction coefficients by the Jennings criterion are underestimated in most cases, while the estimated internal friction coefficients by the new criterion are a little lower in two cases with connectivity rates of 0.5 and 0.6 but a little higher in three cases with connectivity rates of 0.2, 0.4 and 0.8. In general, the results acquired by the new criterion are much better than those by the Jennings criterion for samples with discontinuities of various connectivity rates. It indicates that the errors of results estimated by the new criterion are obviously much lower than those by the Jennings criterion except for connectivity rates of 0.4, 0.5 and 0.6, under which conditions the average errors of results estimated by the two criterions are quite close.

As shown above, the cohesion and the internal friction coefficient estimated by the new criterion can reflect the actual strength parameters reached from laboratory tests much more correctly.

## 4. Discussion

### 4.1. The Nonlinear Features of the Rock Bridge Strength during Progressive Failure

The rock failure has been proved to be a progressive process of crack initiation, propagation and coalesce [29,35]. During the failure process, the strength parameters involving the cohesion and the internal friction coefficient have a nonlinear relation with the plastic strain that is a quantitative index of the damage degree, i.e., the cohesion decreases following a negative exponential function while the internal friction angle increases following a power function [33].

For the Jennings criterion [12], it is assumed that the strength parameters have a linear trend with the connectivity rate that is another quantitative index of the damage degree. However, the linear Jennings criterion has always been doubted that it could not reflect the nonlinear mechanical behavior of a rock mass during progressive failure, and estimate the strength parameters correctly.

In this study, the parameters *A* and *B* are introduced to correct the weight of the cohesion and the internal friction coefficient of rock bridges respectively. The parameters *A* and *B* are found a nonlinear relation with the connectivity rate through fitting the data from laboratory tests, which are expressed as the exponential and negative exponential functions for the data adopted in this study. Moreover, the new shear strength criterion is an empirical criterion and thus the physical meanings of parameters *A* and *B* are just correcting the weight of the cohesion and the internal friction coefficient as the connectivity rate changes.

It should be pointed that the fitting results in Figure 2 are not very good, i.e., *R*^2^ is not high. This is primarily because the data for such type of test are not sufficient enough for its difficulties of sample preparation and experiment implementation, and thus a further research is needed to incorporate many more data. The exponential function, which is not the best fitting one to characterize the correction coefficient *A*, is adopted mainly regarding the previous research of the variation trend of cohesion during the progressive failure process, i.e., an exponential form [33]. Despite of these, the new criterion has an obvious progress compared with the Jennings criterion.

### 4.2. The Influence of Sample Types

**Figure 5:**
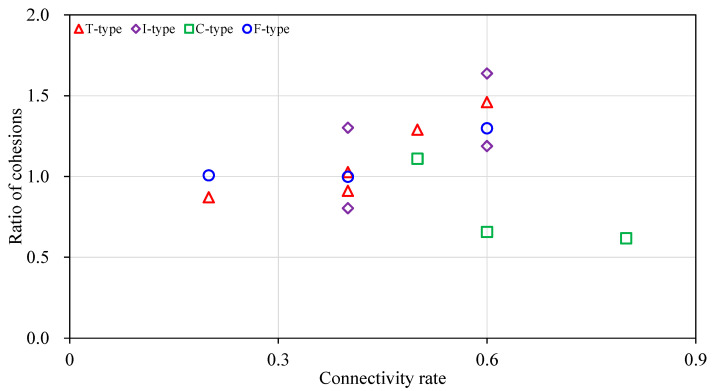
The cohesion ratio of different types of samples.

As mentioned above, the samples were divided into five types according to the positions of non-persistent discontinuities. It has been realized that the fractures can break up the link between particles of a rock and thus weaken the rock cohesion obviously. Therefore, we adopted the estimated cohesion to judge the influence of sample types on the effects of the new shear strength criterion. The relations between cohesion ratios and the connectivity rate of different sample types are shown in Figure 5, in which the red triangles, purple rotating squares, green squares and blue circles denote the results of T-type, I-type, C-type and F-type respectively.

It is interesting that the cohesions generally increase as the connectivity rate increases for most of sample types, i.e., T-type, I-type and F-type but has a decreasing trend for samples of C-type. It means that the criterion may gradually overestimate the cohesions as the connectivity rate increases for samples containing the terminal, intermediate and front discontinuities/discontinuity, but underestimate the cohesions for samples with composite discontinuities. It is a difficult task to judge the validity of such a rule, because it may also result from the influence of various failure modes. The mechanism for these features should be studied further.

## 5. Conclusions

The strength parameters of a rock mass involving the cohesion and the internal friction coefficient have been realized to change nonlinearly during the progressive failure process [33]. However, both the cohesion and the internal friction coefficient have a linear relation with the connectivity rate of rock masses containing non-persistent discontinuities in the commonly used Jennings criterion [12].

In this paper, we collected sixteen groups of test data from the published literatures, and divided the samples to five types according to the positions of discontinuities. After that, a new nonlinear shear strength criterion was put forward by introducing two correction coefficients *A* and *B* into the basic form of the Jennings criterion, which could correct the weight of the cohesion and the internal friction coefficient of rock bridges respectively. The new criterion was determined eventually by fitting the basic form of the Jennings criterion with the laboratory data. It was found that the parameter *A* increased with the connectivity rate and could be expressed as a nonlinear exponential function. On the other hand, the parameter *B* decreased with the connectivity rate and could be described as a nonlinear negative exponential function. It showed that both the cohesion and the internal friction coefficient estimated by the new criterion were superior to those estimated by the Jennings criterion. In comparison with the linear Jennings criterion, there was a better applicability for the new nonlinear shear strength criterion.

## Figures and Tables

**Table 1:** Strength parameters of artificial rock mass samples with various discontinuity positions and connectivity rates.

Sample Type	Connectivity Rate	Quality Ratio of Materials	Cohesion of the Rock Bridge (MPa)	Angle of the Internal Friction of the Rock Bridge (°)	Cohesion of the Discontinuity (MPa)	Angle of the Internal Friction of the Discontinuity (°)	Reference
T-type I-type	0.6	Sand: Gypsum: Water = 3:3:2	4.23	26.55	0	35.2	[15]
T-type I-type	0.4	Cement: Sand: Water = 5:5:2	5.2	56.31	0.19	39.69	[17]
T-type I-type C-type	0.2, 0.4, 0.5, 0.6, 0.8	Cement: Sand: Water = 5:5:2	4.7	59.24	0.63	37.95	[19]
T-type	0.5	Cement: Sand: Water = 2:3:1	3.93	39.5	0	32.3	[25]
F-type I-type B-type	0.2, 0.4, 0.6	Quartz sand: Cement = 1:1	8.3	37.16	1.63	32.8	[21]

**Table 2:** Direct shear test results of artificial rock mass samples with various discontinuity positions and connectivity rates.

Sample Type	Connectivity Rate	Shear Rate	Cohesion (MPa)	Coefficient of the Internal Friction	Reference
T-type	0.6	0.003 mm/s	1.186	0.559	[15]
I-type			1.057	0.573	
T-type	0.4	0.005 mm/s	3	1.4	[17]
I-type			3.4	1.3	
T-type	0.2	0.005 mm/s	3.205	1.836	[19]
T-type	0.4		2.585	1.664	
I-type	0.6		1.9367	1.13	
C-type	0.5		2.259	1.238	
C-type	0.6		3.504	1.29	
C-type	0.8		2.687	0.759	
T-type	0.5	0.005 mm/s	1.423	0.821	[25]
F-type	0.2	1 kN/s	5	0.985	[21]
F-type	0.4		4.9	0.743	
F-type	0.6		3.37	0.787	
I-type	0.4		3.76	0.649	
B-type	0.4		8.2	0.61	

**Table 3:** Correction coefficients *A* and *B* of the strength parameters for rock mass samples with various discontinuity positions and connectivity rates.

Sample Type	Connectivity Rate	*A*	*B*	Reference
T-type	0.6	0.701	0.677	[15]
I-type	0.6	0.625	0.749	
T-type	0.4	0.937	1.187	[17]
I-type	0.4	1.065	1.076	
T-type	0.2	0.819	1.256	[19]
T-type	0.4	0.827	1.358	
I-type	0.6	0.829	0.985	
C-type	0.5	0.827	1.035	
C-type	0.6	1.663	1.262	
C-type	0.8	2.322	0.504	
T-type	0.5	0.724	1.225	[25]
F-type	0.2	0.704	1.412	[21]
F-type	0.4	0.853	1.066	
F-type	0.6	0.720	1.321	
I-type	0.4	0.624	0.861	
B-type	0.4	1.516	0.775	

**Table 4:** Comparison of the cohesions estimated by the Jennings and new criterions.

Sample Type	Connectivity Rate	Test Result of the Cohesion *c* (MPa)	Estimated Cohesion by the Jennings Criterion *c*_1_ (MPa)	Ratio of *c*_1_ to *c* *R*_c1_	Estimated Cohesion by the New Criterion *c*_2_ (MPa)	Ratio of *c*_2_ to *c* *R*_c2_	Reference
T-type	0.6	1.186	1.692	1.427	1.7316	1.46	[15]
I-type	0.6	1.057	1.692	1.601	1.7316	1.6382	
T-type	0.4	3.000	3.196	1.065	2.7347	0.9116	[17]
I-type	0.4	3.400	3.196	0.940	2.7347	0.8043	
T-type	0.2	3.205	3.886	1.212	2.7939	0.8717	[19]
T-type	0.4	2.585	3.072	1.188	2.6551	1.0271	
I-type	0.6	1.9367	2.258	1.166	2.302	1.1886	
C-type	0.5	2.259	2.665	1.180	2.5096	1.1109	
C-type	0.6	3.504	2.258	0.644	2.302	0.657	
C-type	0.8	2.687	1.444	0.537	1.6594	0.6175	
T-type	0.5	1.423	1.965	1.381	1.835	1.2896	[25]
F-type	0.2	5.000	6.966	1.393	5.0373	1.0075	[21]
F-type	0.4	4.900	5.632	1.149	4.8957	0.9991	
F-type	0.6	3.370	4.298	1.275	4.3757	1.2984	
I-type	0.4	3.760	5.632	1.498	4.8957	1.302	
B-type	0.4	8.200	5.632	0.687	4.8957	0.597	

**Table 5:** Comparison of the internal friction coefficients estimated by the Jennings and new criterions.

Sample Type	Connectivity Rate	Test Result of the Internal Friction Coefficient tan*φ*	Estimated Internal Friction Coefficient by the Jennings Criterion tan*φ*_1_	Ratio of tan*φ*_1_ to tan*φ* *R*_f1_	Estimated Internal Friction Coefficient by the New Criterion tan*φ*_2_	Ratio of tan*φ*_2_ to tan*φ* *R*_f2_	Reference
T-type	0.6	0.559	0.623	1.1154	0.5988	1.0711	[15]
I-type	0.6	0.573	0.623	1.0876	0.5988	1.045	
T-type	0.4	1.400	1.232	0.88	1.3199	0.9428	[17]
I-type	0.4	1.300	1.232	0.9477	1.3199	1.0153	
T-type	0.2	1.836	1.491	0.8123	2.0005	1.0896	[19]
T-type	0.4	1.664	1.303	0.7829	1.4186	0.8525	
I-type	0.6	1.130	1.140	1.0088	1.0581	0.9364	
C-type	0.5	1.238	1.208	0.9762	1.2148	0.9812	
C-type	0.6	1.290	1.114	0.8637	1.0581	0.8202	
C-type	0.8	0.759	0.926	1.2195	0.86	1.133	
T-type	0.5	0.821	0.728	0.887	0.7208	0.8779	[25]
F-type	0.2	0.985	0.735	0.7464	0.961	0.9756	[21]
F-type	0.4	0.743	0.713	0.9594	0.757	1.0188	
F-type	0.6	0.787	0.690	0.8763	0.6529	0.8296	
I-type	0.4	0.649	0.713	1.0972	0.757	1.1664	
B-type	0.4	0.610	0.713	1.1673	0.757	1.241	
